# Role of the VEGF-Flt-1-FAK pathway in the pathogenesis of osteoclastic bone destruction of giant cell tumors of bone

**DOI:** 10.1186/1749-799X-5-85

**Published:** 2010-11-09

**Authors:** Yoshihiro Matsumoto, Yuko Okada, Jun-ichi Fukushi, Satoshi Kamura, Toshifumi Fujiwara, Keiichiro Iida, Mihoko Koga, Shuichi Matsuda, Katsumi Harimaya, Akio Sakamoto, Yukihide Iwamoto

**Affiliations:** 1 Department of Orthopaedic Surgery, Graduate School of Medical Sciences, Kyushu University, 3-1-1 Maidashi, Higashi-ku, Fukuoka 812-8582, Japan

## Abstract

**Background:**

Giant cell tumors (GCTs) of bone are primary benign bone tumors that are characterized by a high number of osteoclast-like multinuclear giant cells (MNCs). Recent studies suggest that the spindle-shaped stromal cells in GCTs are tumor cells, while monocyte-like cells and MNCs are reactive osteoclast precursor cells (OPCs) and osteoclasts (OCs), respectively. In this study, we investigated the pathogenesis of osteoclastic bone destruction in GCTs by focusing on the role of the vascular endothelial growth factor (VEGF)-Flt-1 (type-1 VEGF receptor)-focal adhesion kinase (FAK) pathway.

**Methods:**

The motility of OPCs cells was assessed by a chemotaxis assay and the growth of OPCs was examined using a cell proliferation assay. The expression of VEGF and activation of Flt-1 and FAK in clinical GCT samples and in OPCs were detected by immunohistochemistry and immunoblotting. The correlation between the expression levels of activated Flt-1 and FAK and clinical stages of GCTs was investigated by immunohistochemistry.

**Results:**

In GCT samples, CD68, a marker of OPCs and OCs, co-localized with Flt-1. Conditioned media from GCT tissue (GCT-CM) enhanced the chemotaxis and proliferation of OPCs. GCT-CM also stimulated FAK activation in OPCs *in vitro*. Moreover, there was a correlation between the clinical stage of GCTs and the expression of tyrosine-phosphorylated Flt-1 and FAK.

**Conclusions:**

Our results suggest that the VEGF-Flt-1-FAK pathway is involved in the pathogenesis of bone destruction of GCTs.

## Background

Giant cell tumors (GCTs) of bone are rare primary skeletal neoplasms that occur in young adults [1]. The histological phenotype of GCTs is characterized by a large number of osteoclast-like giant multi-nuclear cells (MNCs), which is why this tumor is called an osteoclastoma or giant cell tumor. Apart from the MNCs, GCTs contain two types of mononuclear cells. One cell type has a round morphology and resembles monocytes (monocyte-like cells), while the other is a spindle-shaped, fibroblast-like stromal cell (stromal cells) [2]. Primary cell cultures of GCTs revealed that the stromal cells are likely the proliferating cell type in GCTs because the monocyte-like cells and MNCs are lost after several culture passages [3]. Based on these observations, the current hypothesis for the cellular origin of GCTs is that the stromal cells in GCTs are tumor cells, the monocyte-like cells are reactive macrophages and/or osteoclast precursor cells (OPCs), and the MNCs are reactive osteoclasts (OCs) [4].

Recently, it was reported that these stromal cells secrete several cytokines and differentiation factors, including TGF-β [5], MCP-1[6], RANKL [7] and M-CSF [8]. These soluble factors could function as monocyte chemoattractants and stimulate osteoclast differentiation, suggesting that the stromal cells stimulate blood monocytes to migrate into the tumor tissue and enhance *in situ *osteoclastogenesis, leading to extended osteolysis by OCs.

We previously reported that the vascular endothelial growth factor (VEGF)-Flt-1 (type-1 VEGF receptor)-focal adhesion kinase (FAK) pathway may be involved in the chemotaxis and cell proliferation of OPCs and contribute to arthritic joint destruction [9]. VEGF overexpression has also been associated with the biological aggressiveness of GCTs [10]. Therefore, we hypothesized that the stromal cells in GCTs produce VEGF that recruits OPCs to the neoplastic lesions. In this study, we examined clinical GCT samples in order to determine the possible role of the VEGF-Flt-1-FAK pathway in the pathogenesis of bone destruction in GCTs.

## Methods

### Patients and tissue specimens

The Institutional Review Board of Kyushu University School of Medicine, Fukuoka, Japan approved the protocol to obtain and examine surgical GCT specimens. Twenty-one GCT patients were surgically treated in the Department of Orthopaedic Surgery, Kyushu University. All tumor specimens were formalin-fixed and paraffin-embedded, and 5-mm sections were cut from one representative block for molecular analyses.

### Agents

Sprague-Dawley rats were purchased from KBT Oriental (Saga, Japan). Recombinant human VEGF was obtained from Genzyme/Techne (Minneapolis, MN). Anti-VEGF, -Flt-1 and -Flk-1 Abs were purchased from Santa Cruz Biotechnology (Santa Cruz, CA). The anti-FAK Ab was obtained from Upstate Biotechnology (Lake Placid, NY). Antibodies specific for the phosphotyrosine residue at position 397 in FAK (pY-FAK Ab) and anti-tyrosine phosphorylated Flt-1 (pY-Flt-1) were purchased from Invitrogen (Carlsbad, CA) and Oncogene (San Diego, CA), respectively. The VEGF receptor tyrosine kinase (RTK) inhibitor (ZD4190) was purchased from Calbiochem (San Diego, CA).

### Cell culture

Rat osteoclast precursor cells (rOPCs) were harvested using by the modified method as previously described [11](Takeshita S et al. 2000). Briefly, the femurs and tibias of 1-day-old Sprague-Dawley rats were aseptically resected. The bone ends were cut and the marrow cavity was flushed with α-MEM. The marrow cells were collected, washed and cultured in α-MEM containing 10% FCS and rhM-CSF (100 ng/mL) supplemented with 100 U/mL penicillin and 100 mg/mL streptomycin. After three days of culture, the cells were vigorously washed to remove the nonadherent cells, detached by pipetting and subcultured. After culturing for an additional three days, the cells were harvested and used as rOPCs.

### Immunohistochemistry and immunofluorescence

Immunohistochemistry was performed as previously described [12]. Surgical specimens were initially decalcified for two weeks in an EDTA-containing buffer and embedded in paraffin. The endogenous peroxidase activity was quenched by incubating the sections for an additional 30 min in absolute methanol and 3% hydrogen peroxide. The slides were then incubated with the appropriate primary Abs, followed by biotinylated secondary Abs and peroxidase-conjugated streptavidin. The signals were detected using 3-amino-9-ethylcarbazole in N,N-dimethylformamide. To examine the pY-Flt-1 and pY-FAK levels in GCT samples, the staining intensity of each specimen was scored as follows: 1 (weak staining; less than 10% of cells were positive), 2 (intermediate staining; 10-50% positive) and 3 (strong staining; >50% positive). All molecular variables were scored by one investigator, who was blinded to the clinical stages of the patients.

For immunofluorescence, the samples were incubated with the primary Abs overnight at 4°C. The samples were washed in PBS and then incubated with FITC or TRITC-conjugated secondary Abs. Then, the sections were mounted and examined by confocal laser scanning microscopy.

### Tissue culture of giant cell tumors of bone

Primary cultures of GCTs were obtained from surgical samples of lytic bone lesions. As previously described [6], fresh tumor tissues were minced in DMEM containing 10% FBS supplemented with 100 U/mL penicillin and 100 μg/mL streptomycin. The cell suspension containing small tissue pieces was plated in a 10 cm-culture dish and incubated at 37°C in a humidified atmosphere with 5% CO_2 _and 95% air. Half of the culture medium was replaced every three days with fresh DMEM containing 10% FBS. When the cells reached confluency, the primary cultures were scraped and subcultured. After several passages, the multinucleated giant cells and monocyte/macrophage-like round cells progressively disappeared from the cultures and only the proliferating spindle-shaped cells remained. At passage eight, the cells were cultured with serum-free DMEM for 24 h and the conditioned medium was collected, filtered through 2.5 μm filters, and used as GCT-conditioned medium (GCT-CM).

### Immunoblotting

When the cells reached approximately 70% confluency, they were harvested and solubilized in lysis buffer [20 mM Tris (pH 7.4), 250 mM NaCl, 1.0% NP40, 1 mM EDTA, 50 mg/mL leupeptin, and 1 mM phenylmethylsulfonyl fluoride]. The protein quantity was determined with a Bradford protein assay (Bio-Rad, Hercules, CA). The samples were separated on 4-12% gradient pre-cast MOPS-polyacrylamide gels (Novex, San Diego, CA) and blotted onto nitrocellulose filters. After transfer, the filters were pre-treated with TBS containing 5% dry milk and 0.05% Triton X for 2 h at room temperature and then incubated with the indicated primary antibodies for 2 h at room temperature. After several washes, the membranes were probed with the appropriate horseradish peroxidase-conjugated secondary Abs at room temperature for 1 h. After the final wash, the immunoreactivity of the blots was detected using an enhanced chemiluminescence system (Amersham, Arlington Heights, IL).

### Enzyme-linked immunosorbent assay (ELISA) for VEGF

The VEGF levels in GCT-CM were determined using an ELISA kit from R&D Systems (Minneapolis, MN).

### Cell proliferation assay

rOPCs cells seeded in culture plates were incubated in serum-free media with various reagents (GCT-CM, VEGF and ZD4190) for 24 h. The cell growth rate was determined using a Celltiter-Glo Luminescent Cell Viability Assay Kit (Promega, Madison, WI) according to the manufacturer's protocol.

### Chemotaxis assay

The chemotaxis assay was performed using transwell chambers (Costar, Cambridge, MA) as previously described [13-15]. Briefly, rOPCs were suspended in serum-free α-MEM containing 1% bovine serum albumin and seeded in the upper chamber. The lower chamber was filled with serum-free α-MEM supplemented with or without various cytokines. Polyvinylpyrrolidone-free polycarbonate filters with 8.0-μm pores were coated with type IV collagen and inserted between the two chambers. Then, the cells were allowed to migrate for 6 h at 37°C. After this incubation period, the cells that had migrated to the lower side of the filter were fixed, stained and counted using five fields/filter under a microscope.

### Statistical analysis

The results obtained from the chemotaxis and cell proliferation assays are expressed as the means ± SD and were statistically analyzed by the Student's t-test. The association between the expression levels of various molecular factors (pY-FAK and pY-Flt-1) and the clinical stages were analyzed using the Mann-Whitney U test.

## Results

### Immunolocalization of VEGF, Flt-1 and Flk-1 in GCT samples

**Figure 1:**
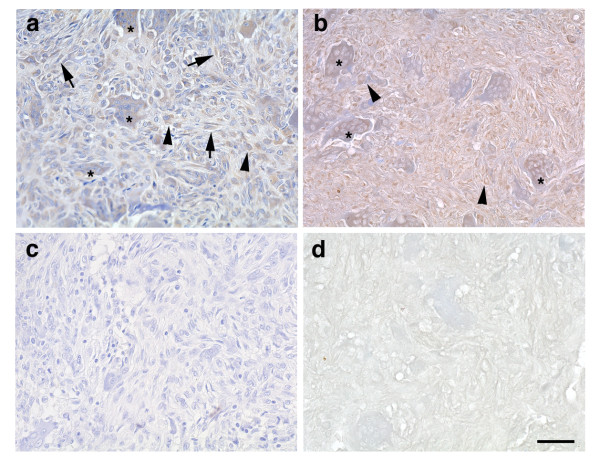
**Expression of VEGF and the VEGF receptors in giant cell tumors (GCTs) of bone**. Surgical specimens were fixed in formalin and serially sectioned. (a) VEGF was expressed in stromal cells (arrows), monocyte-like cells (arrowheads) and multinuclear cells (MNCs) (asterisks). (b) Flt-1 expression was mainly detected in monocyte-like cells (arrowheads) and MNCs (asterisks). (c) Flk-1 expression was not clearly detected in the serial sections. (d) Tissue sections stained with preimmune control IgG showed no specific staining. Original magnification: X 200. Scale bar: 50 μm.

We initially analyzed the expression profiles of VEGF and the VEGF receptors in GCT specimens. TRAP staining demonstrated the presence of bone-resorbing OCs (data not shown). To determine the immunolocalization of VEGF and the VEGF receptors in GCT specimens, we performed immunohistochemistry using serial sections of GCT samples. VEGF expression was observed in all of the stromal cells (arrows), monocyte-like cells (arrowheads) and MNCs (asterisks)(Figure 1a). Flt-1 was expressed in MNCs (asterisks) and a portion of the mononuclear cells that were identified as monocyte-like cells (arrowheads) (Figure 1b). However, Flk-1 expression was not clearly detected in the specimens (Figure 1c). Tissue sections stained with preimmune control IgG showed no specific staining (Figure 1d). These results suggest that Flt-1, but not Flk-1, plays a principal role in VEGF signaling in GCTs.

### Co-localization of CD68 and Flt-1 in monocyte-like cells and MNCs at the site of bone destruction

**Figure 2:**
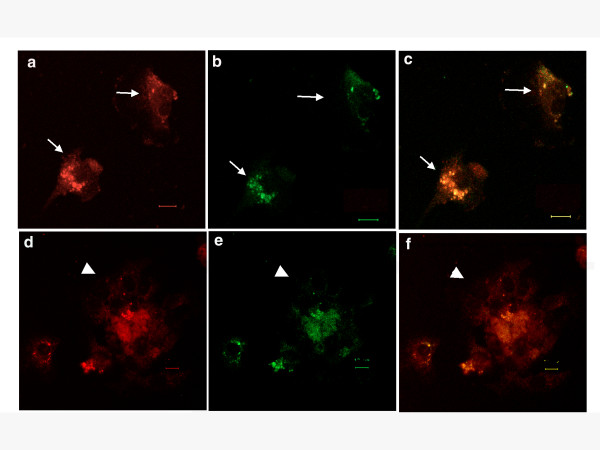
**Co-localization of CD68 and Flt-1 in GCTs**. The sections were prepared as described in Fig. 1 and stained with anti-CD68 (a and d) and anti-Flt-1 (b and e) Abs, followed by TRITC- and FITC-conjugated secondary Abs, respectively. The images were merged (c and f). Arrows indicate monocyte-like cells (a-c) and arrowheads indicate MNCs (d-f). Scale bar: 10 μm.

Because monocyte-like cells and MNCs in GCTs express CD68 [16], we investigated whether Flt-1 co-localized with CD68-positive cells in GCT samples. The specimens were incubated with anti-CD68 (Figure 2a and 2d) and anti-Flt-1 (Figure 2b and 2e) Abs, followed by TRITC- or FITC-labeled secondary Abs, respectively. As shown in Figure 2c and 2f, CD68 and Flt-1 co-localized in monocyte-like cells (arrows) and MNCs (arrowheads) in these specimens. These results suggest that MNCs and monocyte-like cells (thought to be OCs and OPCs, respectively) in the GCT samples expressed Flt-1, indicating that the VEGF-Flt-1 pathway plays specific roles in osteoclastic bone destruction in GCTs.

### Conditioned media from GCT cultures (GCT-CM) enhanced chemotaxis and proliferation of OPCs via VEGF signaling

**Figure 3:**
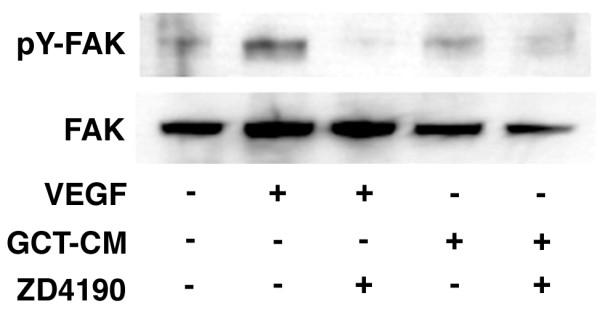
**Effect of GCT-CM on FAK phosphorylation in rat osteoclast precursor cells (rOPCs)**. rOPCs were harvested and cultured in a serum-free medium overnight. Then, the cells were washed and incubated for 5 min in the presence of various reagents (GCT-CM, VEGF and ZD4190). The samples were immunoblotted with anti-tyrosine phosphorylated FAK (anti-pY-FAK) or anti-FAK Abs.

**Figure 4:**
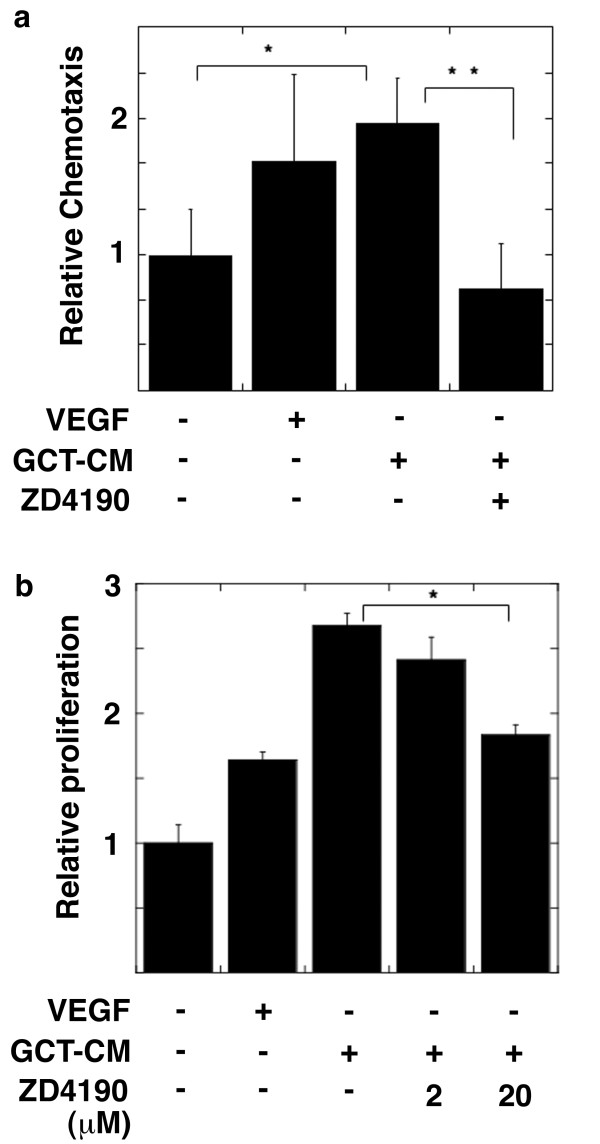
**Effect of GCT-CM on the chemotaxis and proliferation of rat osteoclast precursor cells (rOPCs)**. (a) rOPCs were cultured in serum-free medium overnight and washed twice with PBS. The cells were added to the upper compartment of a modified Boyden chamber. GCT-CM and VEGF (10 ng/mL) with or without ZD4190 were added to the lower compartments, and the chambers were incubated for 6 h at 37°C. The migrated cells were stained and counted as described in the Materials and Methods. The results are shown as the means ± SD of two independent experiments that were performed triplicate (* p < 0.01). (b) rOPCs in a 96-well plate were cultured in a serum-free medium for 24 h and washed with PBS. Then, the cells were stimulated with VEGF and GCT-CM with or without ZD4190 for 24 h. Cellular proliferation was assessed by the Celltiter-Glo Luminescent Cell Viability Assay. Results show the means ± SD of two independent experiments that were performed in triplicate. (* p < 0.01).

Next, we attempted to elucidate whether VEGF-signaling is involved in recruiting CD68-positive cells, such as OPCs, in GCTs. We investigated the effects of GCT-CM on the biological phenotypes of OPCs. To examine the VEGF protein in GCT-CM, we used a VEGF-ELISA, and confirmed that the VEGF concentration in GCT-CM was approximately 2.8 ng/mL. We previously showed that VEGF treatment stimulates the tyrosine phosphorylation of Flt-1 in RAW cells, a model of OPCs [9]. In this study, we used OPCs derived from rat bone marrow cells (rOPCs) [17]. We previously reported that VEGF stimulated the interaction between tyrosine phosphorylated Flt-1 (pY-Flt-1) and FAK, resulting in the autophosphorylation of the tyrosine residue at position 397 in FAK (pY-FAK) in RAW cells. We thus investigated the effects of GCT-CM on pY-FAK in rOPCs and found that GCT-CM increased pY-FAK expression and that this effect was inhibited by ZD4190 treatment (Figure 3). Since we previously reported that VEGF stimulated the chemotaxis and proliferation of RAW cells [9], we investigated the effects of GCT-CM on the chemotaxis and proliferation of rOPCs. GCT-CM enhanced the chemotaxis and proliferation of rOPCs to levels that were comparable to VEGF stimulation, and the addition of ZD4190 to the GCT-CM inhibited these effects (Figure 4a and 4b). These results suggest that GCT-CM enhanced the chemotaxis and cell proliferation of OPCs via VEGF-Flt-1-FAK signaling.

### Possible involvement of the VEGF-Flt-1-FAK pathway in the bone destruction of GCTs

**Figure 5:**
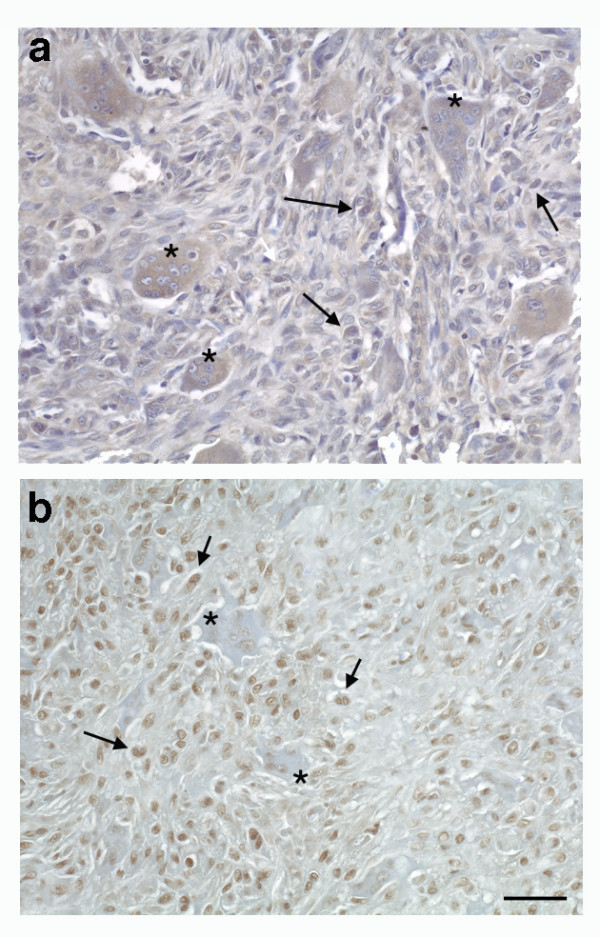
**Expression of pY-Flt-1 and pY-FAK in GCTs**. The sections were prepared as described in Fig. 1 and stained with anti-pY-Flt-1 (a) and anti-pY-FAK (b) Abs. (a) pY-Flt-1 expression was detected in monocyte-like cells (arrows) and MNCs (asterisks). (b) pY-FAK expression was mainly detected in monocyte-like cells (arrows) but not MNCs (asterisks). Original magnification: X 200. Scale bar: 50 μm.

Immunohistochemical analyses showed that pY-Flt-1 was expressed in monocyte-like cells and MNCs (Figure 5a) and that pY-FAK was expressed in monocyte-like cells in GCT specimens (Figure 5b). These results suggest that VEGF binding to its receptor, Flt-1, on monocyte-like cells may induce the tyrosine phosphorylation of FAK in cells within GCTs.

### Correlation between the clinical stage and pY-Flt-1 and pY-FAK expression in GCTs

**Figure 6:**
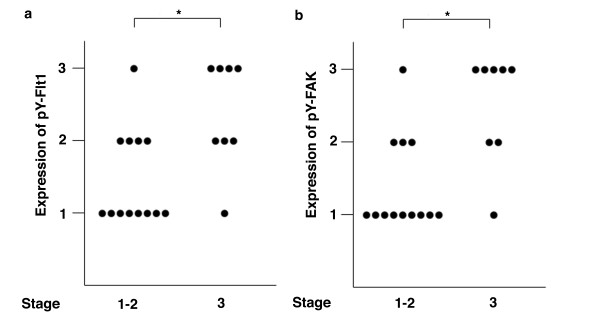
**Correlation between pY-Flt-1 and pY-FAK expression and the clinical stage of GCTs at presentation**. To determine the status of pY-Flt-1 and pY-FAK in GCT samples, the staining intensity of each specimen was scored as follows: 1 (weak staining; less than 10% of the cells were positive cells), 2 (intermediate staining; 10-50% positive) and 3 (strong staining; >50% positive). The Mann-Whitney U test was used to test the significance of the difference between stages I-II and stage III GCTs. The expression of pY-Flt-1 (a) and pY-FAK (b) in stage III GCTs was significantly higher than the expression levels in stages I-II GCTs (p < 0.05). *; p < 0.05 stages I-II versus stage III).

To determine the biological significance of VEGF-Flt-1-FAK signaling in GCTs, we examined the correlation between the expression levels of pY-Flt-1 and pY-FAK and the clinical stages of GCTs. Based on plain X-ray films at the time of presentation, 11 cases were clinically graded as stage II GCTs, eight cases as stage III, and only two cases as stage I. Immunohistochemical analysis showed that the pY-Flt-1 and pY-FAK expression levels in stage I-II GCTs were significantly lower than those in stage III GCTs (p < 0.05) (Figure 6a and 6b).

## Discussion

The association between VEGF expression and angiogenesis has been detected in many solid tumors. In addition, VEGF-induced vascularization during bone development is critical for the formation of OCs [18,19]. Therefore, VEGF may be involved in both angiogenesis and osteoclastogenesis. It has been reported that the level of VEGF gene expression in GCTs correlates with the clinical stage at presentation defined by Enneking's surgical staging system [10], suggesting that the production of VEGF by tumor cells and the induction of angiogenesis may partially contribute to tumor progression. In this study, VEGF was clearly expressed in stromal cells, monocyte-like cells and MNCs in GCTs. CD68, an intracellular glycoprotein, was expressed in monocyte lineage cells, including OPCs and OCs [20]. Therefore, it is possible that the infiltrating MNCs and monocyte-like cells in GCTs mature into OCs and OPCs, respectively. In contrast, the stromal cells did not express CD68, suggesting that they did not originate from the monocyte-macrophage lineage.

In endothelial cells, the VEGF signals were mainly mediated via Flk-1, the type-II VEGF receptor [21]. However, in monocytic lineage cells, most VEGF signals were transmitted via Flt-1, as was previously shown [9]. In regard to the effect of VEGF on monocytes migration, VEGF stimulated the chemotaxis of human monocytes corresponding to the previous report [22]. (Control; 5 ± 1 cells, 10 nM VEGF: 50 ± 5 cells, 10 nM MCP-1: 99 ± 3 cells) We also found that VEGF treatment induced the tyrosine phosphorylation of FAK (pY-FAK). In this study, we first demonstrated that CD68 and Flt-1 co-localized in MNCs and monocyte-like cells, which are thought to be OPCs in GCTs. However, these cells did not express Flk-1. We also indicated that these cells expressed an activated and tyrosine-phosphorylated Flt-1 (pY-Flt-1) as shown in Figure 5. In addition, pY-FAK was expressed in monocyte-like cells in GCT surgical specimens. These results support the hypothesis that VEGF is released from stromal cells, pOCs and OCs. Then, through paracrine and autocrine mechanisms, the secreted VEGF activates the VEGF-Flt-1-FAK pathway. The activation of this signaling pathway might be involved in the migration of these cells into the lesion at the site of bone destruction in GCTs.

We recently showed that VEGF stimulates the chemotaxis and cell proliferation of RAW cells, a model of mouse OPCs. Thus, we investigated the biological effects of VEGF in GCTs using GCT-CM and rOPCs. Consistent with the immunohistochemistry results, GCT-CM contained VEGF and treating rOPCs with GCT-CM resulted in the tyrosine phosphorylation of FAK within cells. GCT-CM also stimulated the chemotaxis and proliferation of rOPCs. All of these GCT-CM-induced effects were inhibited by adding ZD4190, a VEGF RTK inhibitor, to the GCT-CM. It was recently reported that VEGF treatment induces the formation of osteoclasts in osteopetrotic (op/op) mice that lack functional macrophage colony-stimulating factor [23]. Thus, it is possible that the VEGF produced by GCTs directly stimulates the formation of MNCs within the tumor. These results suggest that the effects of GCT-CM, including the stimulation of chemotaxis and proliferation of rOPCs but not osteoclastogenesis, were partially dependent on VEGF-Flt-1-FAK signaling and that this signaling plays important roles in recruiting OPCs into the GCT tissue. On the other hand, ZD4190 did not completely block the basal level of chemotaxis and proliferation of rOPCs. Therefore, we assumed that many other cytokines, including TGF-β1[5], MCP-1[6] and M-CSF, in GCTs influence the chemotaxis and growth of rOPCs. Meanwhile, a recent study showed that GCTs enhanced osteoclastogenesis via paracrine VEGF secretion under local hypoxic conditions and indicated that this might be a critical mechanism for the pathogenesis of GCTs [24]. However, when we harvested GCT-CM under normoxia, GCT-CM did not enhance the osteoclastogenesis of OPCs. Therefore, the role of VEGF in osteoclastogenesis in GCTs *in vivo *should be further investigated.

To assess the pathological significance of the VEGF-Flt-1-FAK pathway, we also examined the correlation between the pY-Flt-1 and pY-FAK expression levels and the clinical stage of GCTs at presentation. The biological aggressiveness of the tumors was classified as previously described [25]. In the present study, we demonstrated that the pY-Flt-1 and pY-FAK expression levels correlated with clinical stage of the tumor. A relatively high level of pY-Flt-1 and pY-FAK expression was observed in stage III GCTs compared with stages I-II GCTs. Although a larger number of tumors are needed to confirm these clinical correlations, our results suggest that activation of the VEGF-Flt-1-FAK pathway may contribute to the clinical progression of GCTs.

## Conclusions

In conclusion, our results suggest that the VEGF-Flt-1-FAK pathway is potentially involved in recruiting OPCs in GCTs. This pathway, in concert with other factors such as TGF-β and MCP-1, may stimulate the recruitment and cell proliferation of OPCs into GCTs, resulting in tumor progression. In this study, ZD4190, a p.o.-active VEGF RTK inhibitor, disrupted VEGF signaling mediated by Flt-1 as well as Flk-1, indicating that ZD4190 administration may simultaneously inhibit VEGF-induced angiogenesis and the recruitment and proliferation of OPCs in GCTs. Therefore, it is conceivable that VEGF RTK inhibitors may be a useful clinical therapeutic for GCTs.

## Competing interests

The authors declare that they have no competing interests.

## Authors' contributions

YM conceived of the study, carried out the experimental studies, and drafted the manuscript. YO, JF, SK, TF, KI and MK carried out experimental studies. SM, KH and AS participated in the design of the study and performed the data analysis. YI participated in its design and helped to draft the manuscript. All authors read and approved the final manuscript.
